# Walk-through flat panel total-body PET: a patient-centered design for high throughput imaging at lower cost using DOI-capable high-resolution monolithic detectors

**DOI:** 10.1007/s00259-023-06341-x

**Published:** 2023-07-19

**Authors:** Stefaan Vandenberghe, Florence M. Muller, Nadia Withofs, Meysam Dadgar, Jens Maebe, Boris Vervenne, Maya Abi Akl, Song Xue, Kuangyu Shi, Giancarlo Sportelli, Nicola Belcari, Roland Hustinx, Christian Vanhove, Joel S. Karp

**Affiliations:** 1 Medical Image and Signal Processing, Department of Electronics and Information Systems, Faculty of Engineering and Architecture, Ghent University, Corneel Heymanslaan 10, 9000 Ghent, Belgium; 2 Division of Nuclear Medicine and Oncological Imaging, Department of Medical Physics, CHU of Liege, Quartier Hôpital, Avenue de Hôpital, 1, 4000 Liège 1, Belgium; 3 Department of Nuclear Medicine, Inselspital, Bern University Hospital, University of Bern, Bern, Switzerland; 4 Dipartimento Di Fisica “E. Fermi”, Università Di Pisa, Italy and with the Instituto Nazionale Di Fisica Nucleare, Sezione Di Pisa, 56127 Pisa, Italy; 5 Physics and Instrumentation, Department of Radiology, University of Pennsylvania, Philadelphia, PA USA

**Keywords:** Positron emission tomography, Total-body PET, Monolithic detector, Patient throughput, Cost-lifetime analysis, Walk-through PET, BGO, L(Y)SO

## Abstract

**Purpose:**

Long axial field-of-view (LAFOV) systems have a much higher sensitivity than standard axial field-of-view (SAFOV) PET systems for imaging the torso or full body, which allows faster and/or lower dose imaging. Despite its very high sensitivity, current total-body PET (TB-PET) throughput is limited by patient handling (positioning on the bed) and often a shortage of available personnel. This factor, combined with high system costs, makes it hard to justify the implementation of these systems for many academic and nearly all routine nuclear medicine departments. We, therefore, propose a novel, cost-effective, dual flat panel TB-PET system for patients in upright standing positions to avoid the time-consuming positioning on a PET-CT table; the walk-through (WT) TB-PET. We describe a patient-centered, flat panel PET design that offers very efficient patient throughput and uses monolithic detectors (with BGO or LYSO) with depth-of-interaction (DOI) capabilities and high intrinsic spatial resolution. We compare system sensitivity, component costs, and patient throughput of the proposed WT-TB-PET to a SAFOV (= 26 cm) and a LAFOV (= 106 cm) LSO PET systems.

**Methods:**

Patient width, height (= top head to start of thighs) and depth (= distance from the bed to front of patient) were derived from 40 randomly selected PET-CT scans to define the design dimensions of the WT-TB-PET. We compare this new PET system to the commercially available Siemens Biograph Vision 600 (SAFOV) and Siemens Quadra (LAFOV) PET-CT in terms of component costs, system sensitivity, and patient throughput. System cost comparison was based on estimating the cost of the two main components in the PET system (Silicon Photomultipliers (SiPMs) and scintillators). Sensitivity values were determined using Gate Monte Carlo simulations. Patient throughput times (including CT and scout scan, patient positioning on bed and transfer) were recorded for 1 day on a Siemens Vision 600 PET. These timing values were then used to estimate the expected patient throughput (assuming an equal patient radiotracer injected activity to patients and considering differences in system sensitivity and time-of-flight information) for WT-TB-PET, SAFOV and LAFOV PET.

**Results:**

The WT-TB-PET is composed of two flat panels; each is 70 cm wide and 106 cm high, with a 50-cm gap between both panels. These design dimensions were justified by the patient sizes measured from the 40 random PET-CT scans. Each panel consists of 14 × 20 monolithic BGO detector blocks that are 50 × 50 × 16 mm in size and are coupled to a readout with 6 × 6 mm SiPMs arrays. For the WT-TB-PET, the detector surface is reduced by a factor of 1.9 and the scintillator volume by a factor of 2.2 compared to LAFOV PET systems, while demonstrating comparable sensitivity and much better uniform spatial resolution (< 2 mm in all directions over the FOV). The estimated component cost for the WT-TB-PET is 3.3 × lower than that of a 106 cm LAFOV system and only 20% higher than the PET component costs of a SAFOV. The estimated maximum number of patients scanned on a standard 8-h working day increases from 28 (for SAFOV) to 53–60 (for LAFOV in limited/full acceptance) to 87 (for the WT-TB-PET). By scanning faster (more patients), the amount of ordered activity per patient can be reduced drastically: the WT-TB-PET requires 66% less ordered activity per patient than a SAFOV.

**Conclusions:**

We propose a monolithic BGO or LYSO-based WT-TB-PET system with DOI measurements that departs from the classical patient positioning on a table and allows patients to stand upright between two flat panels. The WT-TB-PET system provides a solution to achieve a much lower cost TB-PET approaching the cost of a SAFOV system. High patient throughput is increased by fast patient positioning between two vertical flat panel detectors of high sensitivity. High spatial resolution (< 2 mm) uniform over the FOV is obtained by using DOI-capable monolithic scintillators.

## Introduction

Different total-body (TB) PET systems have been developed during the last years [1] that was initiated through an NIH grant forming the EXPLORER consortium with UC Davis and UPenn. This resulted in development of a research scanner, the PennPET Explorer at UPENN with axial FOV ranging from 64 to 142 cm [2, 3] and a commercial product by United Imaging, the uEXPLORER with axial FOV of 194 cm [4]. A second commercial product was released soon after by Siemens, the Vision Quadra with axial FOV of 106 cm [5]. These TB-PET systems have now been installed in a reasonable number (> 25) of large centers in Europe, Asia, Australia, and the USA (mostly in academic sites of which some are supported by grant money). The acquisition and installation of most of these TB-PET scanners have at least partially been funded by grant money. This shows the large research potential of such long axial field-of-view (LAFOV) PET systems (e.g., studies on low radiation–dose imaging, TB dynamic imaging, new radiotracer development, delayed imaging, and brain–body interactions) [6–9]. For clinical routine imaging in more standard nuclear medicine (NM) centers (or for academic (research) centers with limited access to large grants), the high acquisition and service costs are, however, an obstacle as it is very hard to convince hospitals that these costs will be compensated (balanced) by an increased income for the NM department. These systems have about four to eight times the number of detectors of a standard PET-CT, which leads to an increase in system cost for the PET roughly linearly correlated with the increase in detectors [1]. At the time of writing, the sales price of a standard axial field-of-view (SAFOV) PET-CT is about 2–3 MEuro, resulting in a cost of about 8–12 MEuro for TB-PET scanners with 1–2 m LAFOVs.

The number of required PET scans continues to rise at an estimated rate of about 11% per year [10], and we also observe an increasing demand for PET-CT scans in the evaluation and follow-up for radionuclide, chemo-, radio-, and immuno-therapy. Besides the growth of 2-deoxy-2-[^18^F]fluoro-d-glucose ([^18^F]FDG) PET in the field of oncology, there is also an increase in the number of scans with more disease-specific tracers, like the radiolabeled prostate–specific membrane antigen (PSMA) ligands. Furthermore, the number of screening methods to enable disease detection at earlier stages (e.g., based on blood sampling, genetic tests or patient history) is also expanding. Lung cancer screening with CT (heavy long-term smokers) is currently being tested in the USA and Europe [11]. More recently, the EU has also released a new approach on cancer detection: “screening more and screening better” [12]. Earlier detection will likely lead to improved therapy outcomes and this will lead to an increasing demand for follow-up scans in the future [13]. Imaging after screening can be expected for breast cancer, lung cancer, and prostate cancer [14, 15]. In other words, PET imaging is not only about the detection of tumor and its spread anymore (like it used to be) but plays more and more an essential role in early diagnosis based on selected screening approaches [6]. To be able to follow this trend (and satisfy the clinical needs) without further increasing healthcare (imaging) costs, NM departments will require the capability to scan more patients. Increased patient throughputs can be achieved with faster imaging (acquisition) times, and ideally this can be realized with lower cost imaging systems and with less personnel per scan (often a limiting factor in NM departments) [1].

When we look at the current systems, TB-PET easily fulfills the fast PET imaging (and/or low radiation dose) requirement [6]. Good-quality static PET scans of the torso at standard radiotracer injected activity (typically around 2.5 MBq/kg for [^18^F]FDG, maximum 370 MBq) can be obtained on a TB-PET in a 30-s to 2-min PET acquisition time. However, the full system still suffers from quite low effective patient throughput (at least 10 min per patient), where the limiting factor is the patient positioning on/off the bed and, to a lesser extent, the time to obtain the required scout view and CT. Nowadays, combined PET-CT dual-modality imaging has almost become indispensable in clinical NM routine; the low-dose CT is not only used for anatomical co-registration with the PET image but also offers an easy and fast way to perform attenuation and scatter correction in PET.

In this paper, we first present a patient-centered design of a dual-panel TB-PET setup based on high-resolution monolithic detectors and compare its performance in terms of component costs, sensitivity, patient throughput, and required dose per patient with SAFOV and LAFOV PET systems.

## Methods

This section outlines our conceptual design approach for the proposed WT-TB-PET scanner and provides the rationale behind the design choices. The component cost and sensitivity of this new design are compared to existing SAFOV and LAFOV PET systems. Based on patient throughput measurements (performed on two Siemens Biograph Vision 600 PET-CTs and the WT-TB-PET mock-up) and sensitivity values (determined from GATE simulations), we extrapolate the PET acquisition time required to obtain an equal image quality (at an equal radiotracer injected activity) and compare the patient throughput times of a LAFOV scanner (in limited and full acceptance mode) and the new WT-TB-PET design.

### Design of the walk-through total-body PET

#### Design concept and system dimension

The WT-TB-PET concept has similarities with recent flat panel airport scanners for passenger security inspection. The patient stands between two flat panels and is scanned directly (within seconds) after positioning themselves inside the machine on indicated marks. A similar design approach as the Siemens Quadra (with an axial FOV of 106 cm) was followed for determining the axial length: most PET-CT scans are used to image the patient’s torso (and sometimes the head), while the legs are less relevant for about 80–90% of the scans. Axial FOV longer than 1–1.2 m may be interesting for research purposes, but here we focus on the design of a cost-efficient clinical system, and thus, only imaging the torso (including the head) simultaneously is of interest. To compensate for variable patient height, limited vertical movement (max about 30 cm) can be obtained using a patient platform (like in the EOS system which is a commercial system delivering low-dose stereo radiographic images of patients in standing position, https://www.eos-imaging.com/). To accommodate the patients needing a head-to-toe scan, the detector panels need to move upwards and downwards to image the legs/feet in a second position. Alternatively, sequential scanning of the legs is also possible by continuously moving the detector arrays vertically. The number of applications where a total-body scan (simultaneously imaging the leg/feet, torso, and head) is required seems (for now) very limited.

**Fig. 1:**
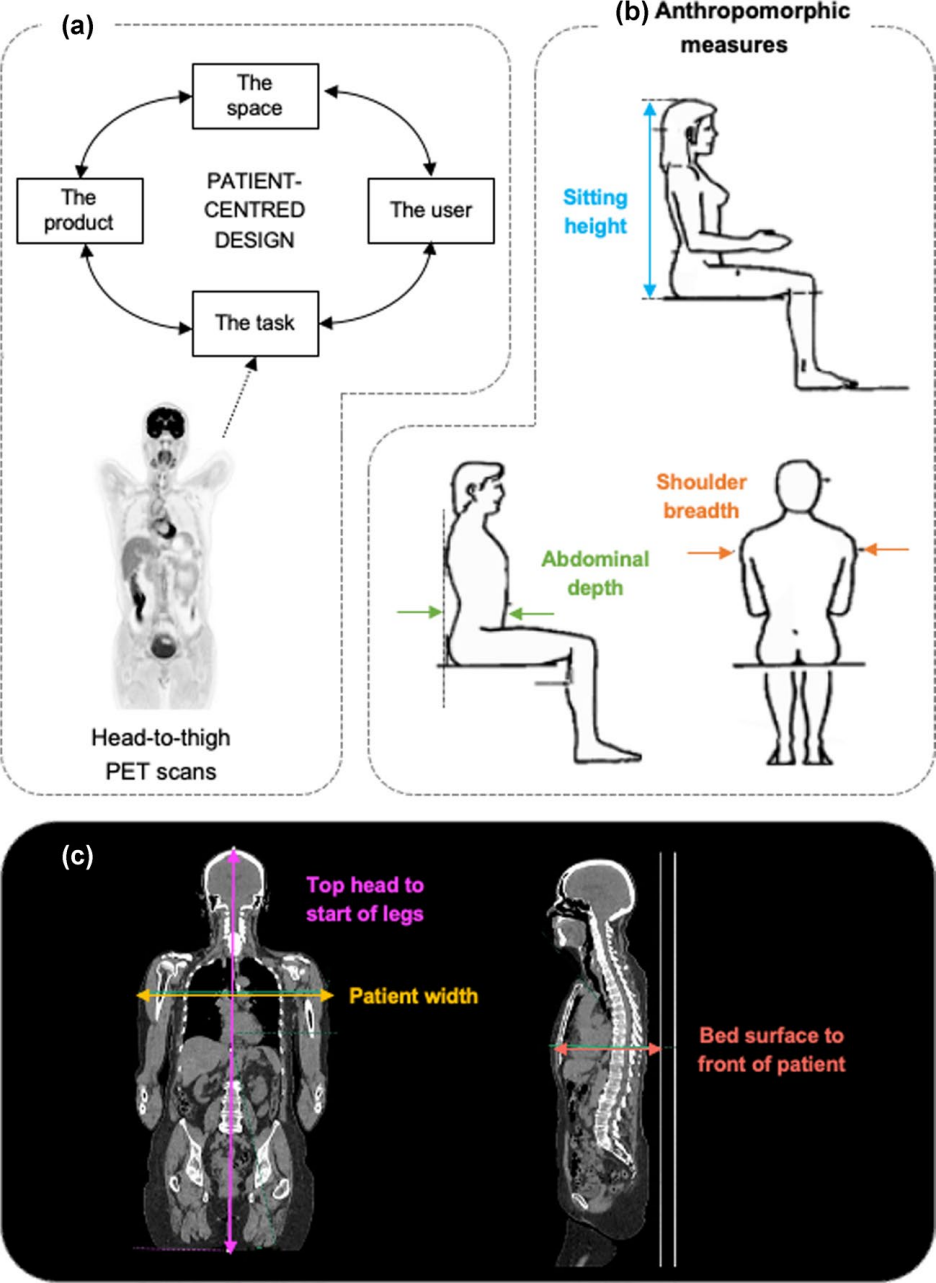
(**a**) System design concept. (**b**) Anthropomorphic measures. (**c**) Measures from CT of PET-CT patients

To determine the dimensions of the WT-TB-PET (and thus also the size of the mock-up that we built), we referred in a first phase to anthropomorphic measures given by the book “Body Space: Anthropometry, Ergonomics, and The Design of Work” [16]. The concept of this book is to provide guidance on the design of products which should be based on the task, considering the space, and the user (see Fig. 1(a)). These must be in balance with each other and adapted optimally to each other. An anthropometric database is also provided in the book, and based on these metrics (i.e., 95% percentile of patient shoulder width, patient depth, and sitting height (= head-to-thigh), Fig. 1(b)), we obtained a first estimate of the required system size. However, PET-CT patients may differ from the general population, especially the abdominal depth may vary with patients being heavier on average and/or having less straight position (e.g., Bechterew). In a next step, we therefore analyzed the PET-CT scans of 40 randomly selected patients from the database at the University Hospital (CHU) of Liège (Belgium). Three measures of patient sizes were directly determined from the CT scans (Fig. 1(c)): top of head to start of the legs, patient width (at the level of shoulders), and distance from PET system bed surface to the front of the patient.

#### Flat panel detector design and detector choice

The detectors currently used in all clinical PET-CT systems are based on pixelated detectors (typically L(Y)SO or BGO-based; 3–4 mm^2^ in size and 20 mm thick) and do not measure depth-of-interaction (DOI). Monolithic detectors (also based on L(Y)SO and BGO crystals) are valid alternatives to pixelated detectors as they have a much higher spatial resolution, are capable to encode DOI information and have a coincidence timing resolution between 200 (LYSO) and 400 ps (BGO) using advanced deep learning methods. Carra et al*.* [17] have shown that a neural network-enhanced readout can achieve < 16% energy resolution with BGO. Monolithic detectors have extensively been used in commercial small animal systems for 5 years (based on developments in our research group in Ghent, Belgium [18]) and are now ready for implementation in clinical systems [19, 20]. The introduction in clinical systems from the big vendors has not happened yet, but clinical systems in smaller companies (like Oncovision) are already equipped with these detectors [21].

The idea with the proposed flat panel PET design is to bring the detectors as close as reasonably possible to the patient (in an upright position) with a gain in both sensitivity and spatial resolution (derived from reduced acolinearity). The chosen detector for the WT-TB-PET is a 16-mm thick 50 × 50 mm^2^ (BGO or LYSO) detector read out by an 8 × 8 array of 6 × 6 mm SiPMs with a 6-layer DOI measurement, developed in the UTOFPET project (University of Pisa and Ghent University) [22]. The choice of detector thickness and transverse size is based on existing preclinical monolithic detectors [18] but scaled up by a factor of 2 for implementation in the WT-TB-PET (clinical system). Thanks to the application of AI techniques [19, 20], this detector has a 1.3-mm intrinsic spatial resolution and a time-of-flight (TOF) kernel of 327 ps (Cherenkov Effect). This bench measurement represents the potential to achieve excellent spatial and timing resolution with a monolithic detector, but for further calculations in this manuscript we assume a conservative 600 ps TOF system resolution for our BGO-based design and 300 ps for our LYSO design.

### Component cost comparison of WT-TB-PET with existing SAFOV and LAFOV systems

A standard (25 cm axial FOV) PET-CT scanner sells nowadays for about 2–3 MEuro. Typically, materials costs are about one-third to one-half of the sales price in this industry. The most expensive components in a PET-CT system are the scintillators, the photosensors (Silicon Photomultipliers, SiPMs nowadays), and the electronics readout. Scintillator material (mostly L(Y)SO) contributes to about 40% of the scanner cost, electronics, and SiPMs around 30%, and the CT scanner adds to about 20% of the cost (depending on the number of slices) [1]. BGO is about 30% of the cost of LSO for the same volume (number obtained from several quotes of vendors), has higher stopping power but in general it has no TOF. This has recently changed due the higher efficiency of SiPMs which enables moderate TOF (400–800 ps) based on Cerenkov light. Based on these numbers and assuming an equal cost for SiPMs per surface area, we conducted a total component cost (scintillator + SiPM material cost) comparison between the new WT-TB-PET with monolithic BGO or LYSO detectors and existing SAFOV and LAFOV systems.

### Comparison of patient throughput estimates

By considering sensitivity, differences in TOF and setup/scout/CT/transfer time, we compared the throughput (total number of patients) of a LAFOV scanner (in limited and full acceptance mode) and the new WT-TB-PET design, assuming the same image quality (and equal radiotracer injected activity). The methodology to derive patient throughput estimates for each scanner design (WT-TB-PET and LAFOV) is explained below.

The sensitivity of the WT-TB-PET is estimated using Monte Carlo simulations with GATE and compared to numbers obtained from literature on the SAFOV and LAFOV systems. To compensate for the high acquisition costs, one needs a sufficiently large number of patients per day (typically 15–30 per scanner in most sites). To estimate the expected possible throughput, we performed time measurements during 1 day on two Siemens Biograph Vision 600 PET-CTs in the clinical NM department at the CHU of Liège (Belgium), which has recently optimized its imaging workflow. From those measurements, we obtained the average duration of the PET acquisition, dependent on the clinical investigation (extent of the field of view), body mass index (BMI), on which PET acquisition speed is adapted, the height of the patient, the patient set-up and transfer time, as well as the duration for scout and CT scans. The expected throughput on a LAFOV scanner from the same manufacturer (Siemens Quadra) was estimated by compensating for the higher sensitivity of the system (in both limited angle and full axial acceptance mode) with a reduction in the PET scan time. TOF is the same on both systems, so no compensation is required. We focussed here on sensitivity for longer object like torso (so-called NEMA or line sensitivity), the main application in PET. Both volume and point sensitivity matter and are intertwined: when imaging an organ + torso in a total-body PET scanner, we can spend more time on organ because we do not axially translate the bed. When imaging a single organ (e.g., brain) we typically see a smaller gain (more 3–4 ×) for LAFOV PET systems.

**Fig. 2:**
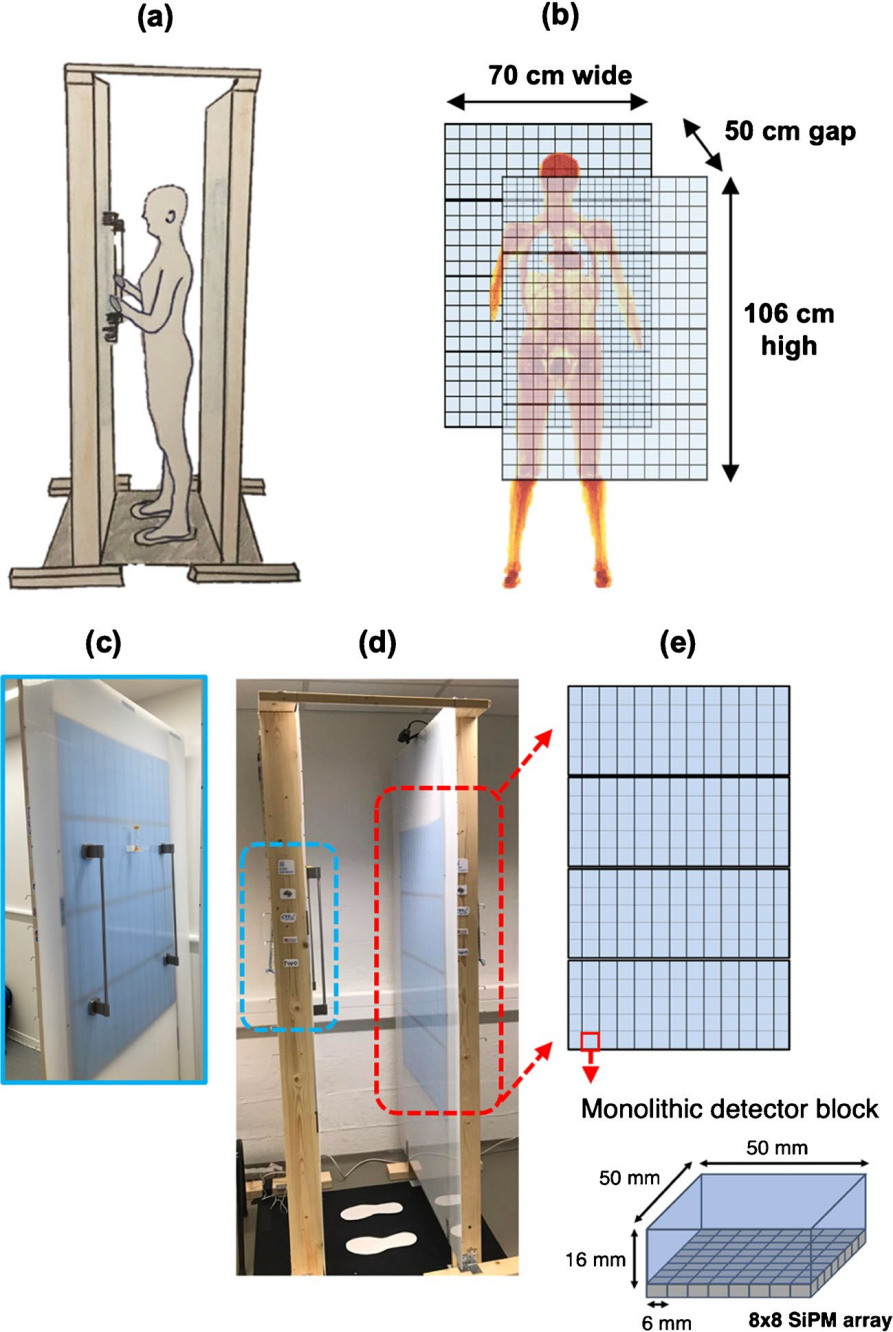
(**a**) Artist view of the walk-through TB-PET. (**b**) Flat panel dimensions and design. (**c**) One side of the mock-up with the handlebars installed to reduce the body motion. (**d**) Side view of the WT-TB-PET mock-up used for patient throughput measurements. Prints of feet are used to let the patient position themself between the flat panels. (**e**) The four blue modules on each panel side can manually be adjusted to the right height

The patient throughput for the WT-TB-PET was estimated in a realistic setting inside the NM department (CHU Liège, Belgium) using the WT-TB-PET mock-up (Fig. 2). In total, 20 patients were asked to position themselves between the two flat panels of the mock-up and stand still for 30 s (estimated acquisition time). Throughput times (door-to-door) were recorded, and based on this, a setup/scout/CT/transfer time was estimated. Of interest to note is that on the day of the measurements with the WT-TB-PET mock-up, four patients were not eligible to participate as one was bedridden and three were in a wheelchair. The extent of motion, potentially larger for standing patients, was tested in a mock-up of the WT-TB-PET and compared to motion of patients lying on a conventional Biograph Vision PET-CT (CHU Liège). Using a Microsoft Kinect camera, motion of infrared markers placed on subjects’ shoulders, head, chest, and abdomen was tracked.

### Radiotracer cost comparisons

Besides faster imaging and higher patient throughput, there is also a potential for this new WT-TB-PET system to reduce expensive radiotracer costs (especially in cases with more disease-specific and more expensive radiotracers than [^18^F]FDG). In other words, scanning more patients (assuming an equal administered activity of 185 MBq per patient) on the same day impacts the amount of radiotracer activity needed at the beginning of the day. The total radiotracer activity required to scan the estimated number of patients (per system) was then determined, and the required average radiotracer activity per patient at the beginning of the scan was estimated based on the assumption that 1850 MBq remained available at the end of the day. This 1850 MBq should be seen as a backup to cover any potential delays due to patient/scanner problems. We compared those radiotracer dose and cost estimates between the WT-TB-PET, SAFOV, and LAFOV systems.

## Results

### Design description of the walk-through total-body PET

#### Design concept and system dimension

Table 1 presents the measures taken from the anthropomorphic estimates for British Adults [16]. These estimates give a first indication of the required length, width, and gap of a flat panel TB-PET system. Table 2 reports the measurements recorded from 40 CT scans to determine the average and maximum patient size values. Note that the measures directly taken from the CT scan are not representative of the previous anthropomorphic estimates in Table 1. The most comparable measure is the top of the head to the start of the legs, which is equal to the sitting height. Patient width is, on average higher than the shoulder breadth due to the positions of elbows when arms are raised or positioned alongside the body. The distance from the patient’s bed surface to the front of the patient is larger than the abdominal depth as the patient may not completely lie down on the bed.

**Table 1:** Three anthropomorphic measures relevant for the design dimensions of the walk-through TB-PET system are reported as 50^th^ and 95^th^ percentile in the general population [16]

	50%ile	95%ile
Sitting height	910 mm	965 mm
Shoulder breadth (bideltoid)	465 mm	510 mm
Abdominal depth	270 mm	325 mm

**Table 2:** Walk-through TB-PET design dimensions are based on patient sizes as determined from CT scans of 40 randomly selected PET-CT patients

	Mean	Max
Top of head to start of the legs	850 mm	950 mm
Width of the patient	520 mm	650 mm
Patient bed surface to front of the patient	320 mm	380 mm

Based on these values, we propose a flat panel WT-TB-PET system design of 106 cm height, 70 cm width, and a 50-cm gap between the two panels. A first mock-up version with these design dimensions was built (Fig. 2 [23]). A first quick test of the mock-up with volunteers from our department (Ghent University, Belgium) indicated that motion is minimized if the subject can hold on to handlebars and focus while being scanned. Note that a future upgrade will also accommodate bedridden and wheelchair-bound patients.

In the mock-up used for initial patient tests, the panel adjustment is made manually (in 10-cm steps accommodating 1.50–2-m tall patients). Note that the actual scanner will automatically adjust the height of the panels so that the top of the panels is always 10 cm higher than the top of the patient’s head.

#### Flat panel detector design and detector choice

**Fig. 3:**
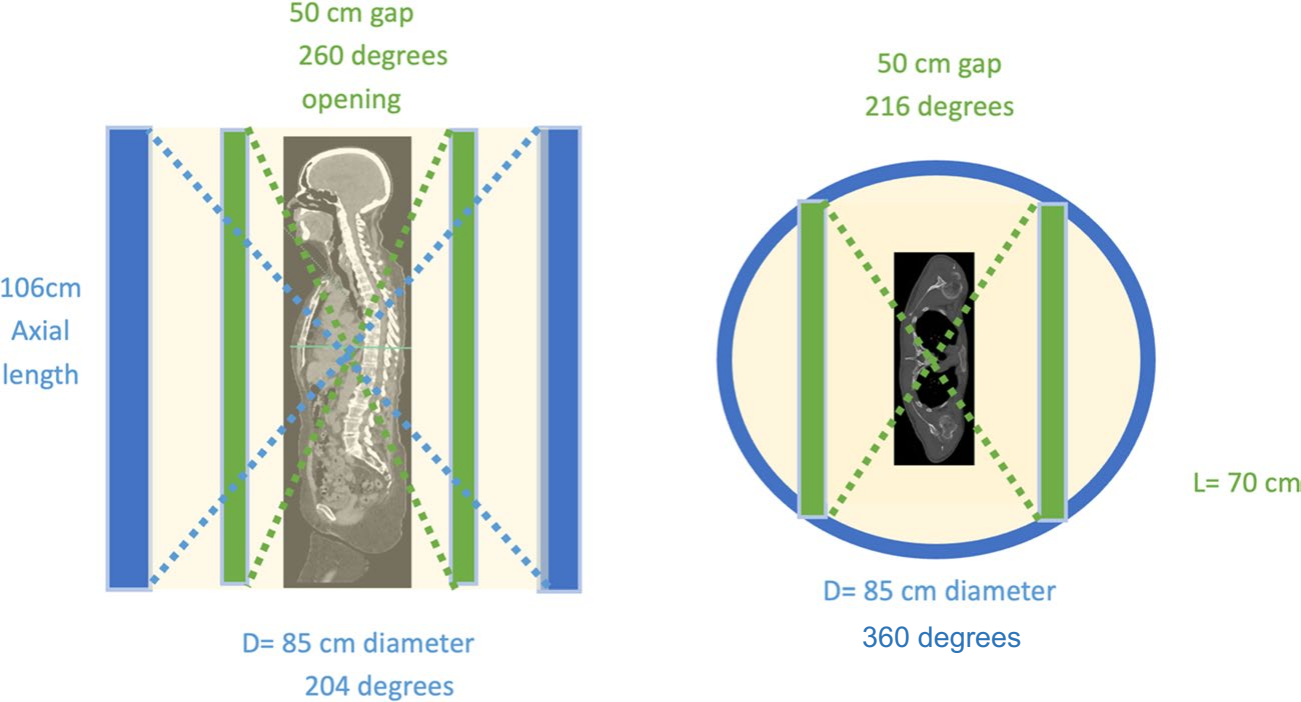
Comparison between the Siemens Vision Quadra design in blue and the walk-through TB-PET design in green. The angles of 204° and 360° show the fraction of the full 360° geometrically captured (axial and transverse) by the LAFOV system. The angles of 260° and 216° show the fraction of the full 360° geometrically captured (axial and transverse) by the WT-PET system

The advantage of a flat panel design is that the detectors are used more efficiently. For the same solid angle, less detectors are required in the WT-TB-PET design as the detectors are positioned much closer to the patient (Fig. 3). The opening angle (of the full 360°) in axial and transverse direction is shown in this figure for both Quadra and our WT-PET design. The number of detectors (for the same axial FOV of 106 cm) is, therefore, 1.9 × less than in a Siemens Quadra (LAFOV PET) with comparable sensitivity. This factor alone reduces the cost of the scanner by a factor of 1.9 (assuming the same detector and readout technology), which brings the cost of a flat panel design almost halfway between current SAFOV and LAFOV systems (see next “Component cost comparison of WT-TB-PET with existing SAFOV and LAFOV systems”).

### Component cost comparison of WT-TB-PET with existing SAFOV and LAFOV systems

Given that the major component cost in a PET system is that of the scintillator and the BGO-based detectors are 3 × lower in cost compared to L(Y)SO, the proposed WT-TB-PET system with a 16-mm thick 50 × 50 mm^2^ BGO detector is at a much lower cost compared to that of the LAFOV with L(Y)SO. Table 3 illustrates the component costs for a standard 26 cm PET-CT (SAFOV), a cylindrical TB-PET with 4 times the axial length (LAFOV = 106 cm, representative of the axial FOV of the Siemens Quadra), and the WT-TB-PET based on 16-mm thick BGO and LYSO. Note that for the analysis, we only compared the cost of the PET system. For SAFOV and LAFOV PET-CT designs, the cost of the CT component should be added and depends on the type of CT chosen. Gaps between pixels and modules are not considered but will probably be reduced in a monolithic block design compared to a pixelated system. SAFOV and LAFOV geometries are modeled after the Vision and Vision Quadra, but not meant to be those systems since then the cost of components becomes more complicated.

**Table 3:** PET component cost analysis (including the scintillator, SiPM, and electronics) comparing a standard PET-CT (SAFOV), a conventional 106 cm TB-PET-CT (LAFOV), and the proposed walk-through TB-PET system. Only the costs of the PET part are considered. The cost of the electronics was not calculated as it is harder as it is harder to estimate and depends strongly on the chosen detector concept and the amount of channels. Based on our first estimates the electronics of our design will be lower cost than for a full pixelated LAFOV scanner

	Scintillator	SiPM	PET component cost
Component cost	L(Y)SO: 30 Euro/cc BGO: 10 Euro/cc	1000 Euro/module One module: 5 × 5 cm surface (8 × 8 array of 6 × 6 mm SiPM)	
SAFOV (85 cm diameter, 20-mm-thick LSO, 26-cm axial FOV)	π × 85 cm × 2 cm × 26 cm × 30 Euro/cc = 416 kEuro	π× 85 cm × 26 cm/ (5 cm × 5 cm) × 1000 Euro = 278 kEuro	694 kEuro
LAFOV (85 cm diameter, 20 mm thick LSO, 4 × 106 cm axial FOV)	π × 85 cm × 2 cm × 26 cm × 4 × 30 Euro/cc = 1666 kEuro	π × 85 cm × 4 × 26 cm/ (5 cm × 5 cm) × 1000 Euro = 1112 kEuro	2776 kEuro
WT-TB-PET (2 panels of 70-cm width each, 106-cm axial FOV, 16-mm-thick BGO)	2 × 70 cm × 106 cm × 1.6 cm × 10 Euro/cc = 237 kEuro	2 × 70 cm × 106 cm/(5 cm × 5 cm) × 1000 Euro = 593 kEuro	830 kEuro
WT-TB-PET (2 panels of 70 cm width each, 106 cm axial FOV, 16-mm-thick LYSO)	2 × 70 cm × 106 cm × 1.6 cm × 30 Euro/cc = 711 kEuro	2 × 70 cm × 106 cm/ (5 cm × 5 cm) × 1000 Euro = 593 kEuro	1304 kEuro

Based on the cost analysis presented in Table 3, the estimated component cost for the proposed WT-TB-PET system with 16-mm-thick monolithic BGO/6 mm SiPMs is only 1.2 × higher than that of a SAFOV and 3.3 × cheaper than that of a LAFOV. For a WT-TB-PET system with 16-mm-thick monolithic LYSO/6 mm SiPMs, the estimated component cost for the proposed WT-TB-PET system with 16-mm-thick monolithic BGO/6 mm SiPMs is only 1.89 × higher than that of a SAFOV and 2.12 × cheaper than that of a LAFOV to estimate and depends strongly on the chosen detector concept and the amount of channels. Based on our first estimates the electronics of our design will be lower cost than for a full pixelated LAFOV scanner.

### Comparison of patient throughput estimates

On a recent SAFOV scanner (Siemens Biograph Vision 600, 26 cm axial FOV, 214 ps), routine clinical [^18^F]FDG scans are done with an average PET-only acquisition time of 10 min for torso imaging (with 2.5 MBq/kg injected activity and a system sensitivity of 16.3 kcps/MBq) [24]. The LAFOV PET version of that Siemens system, the Siemens Quadra (AFOV = 106 cm) has a sensitivity of 83.4 kcps/MBq [4] (in limited ring difference mode) so about 5.1 × higher sensitivity compared to the SAFOV Siemens scanner (translating into 5.1 × shorter acquisition). A recent upgrade of the Quadra uses a full acceptance angle mode which allows to further increase the sensitivity up to 176.0 kcps/MBq [4] (12 × higher than Siemens Biograph Vision 600). While there may be some disadvantages to the full acceptance mode (higher scatter/randoms fraction), it can reasonably achieve 10 × shorter PET acquisition time than the Siemens Biograph Vision 600 (at equal dose). The sensitivity of the WT-TB-PET (152 kcps/MBq) was obtained with Monte Carlo simulation in GATE and included all line-of-responses (LORs). There is no reason to exclude oblique angles (i.e., operation in full acceptance mode) as the system has multiple layers of DOI, resulting in no resolution degradation with oblique angles. Required PET acquisition times are scaled based on the sensitivity differences (Table 4).

**Table 4:** Estimates of throughput and number of patients based on TOF, sensitivity non-PET imaging time

System concept	SAFOV	LAFOV	LAFOV	WT-TB-PET	WT-TB-PET
Axial length (AFOV)	26 cm	106 cm	106 cm	106 cm	106 cm
Scintillator material	LSO	LSO	LSO	BGO	LYSO
Ring differences	All rings	Max 85	All rings	All rings	All rings
	Calculation of PET-only scan time, accounting for sensitivity and TOF				
NEMA sensitivity (kcps/MBq)	16.4	83.4	176.0	152	114
PET scan time (s) based on sensitivity	600	117	55	64	86
TOF resolution (s)	210	230	230	600	300
PET scan time (s) based on sensitivity and TOF gain	600	117	55	182	122
	Calculation of non-PET imaging time, accounting for scan time of scout + CT, positioning, and transfer time				
CT + scout scan time (s)	60	60	60	60	60
Patient scanner positioning (s)	120	120	120	30	30
Patient transfer time (s)	240	240	240	120	120
Non-PET imaging time (s)	420	420	420	210	210
Total time (min) per scan	17	8	7	6,5	5,5
# Scans on 8 h working day	28	53	60	73	86

TOF resolution is expected to be around 400–800 ps at the system level for the WT-TB-PET. Since SAFOV and LAFOV use the same detector technology, the TOF resolution of the Siemens Vision and Quadra system are close to each other, 210 and 230 ps respectively. Note that TOF increases the effective sensitivity: so typically, 2 × less counts are needed when the TOF goes down from 400 to 200 ps to reach the same image quality. The reduced TOF resolution (300 or 600 ps) of the WT-TB-PET (compared to 210 ps for SAFOV and 230 ps for LAFOV) is considered by scaling up the required PET scan time by a factor of 1.9 (ratio of the TOF differences).

The time required for acquiring the scout and CT, patient positioning, and patient transfer (obtained from measurements conducted on the Siemens Biograph Vision 600 at CHU Liège) are summed into the non-PET imaging time. Comparing the SAFOV and LAFOV, these non-PET imaging times are expected to be the same. The total scan time is then the sum of the PET scan time and the non-PET imaging time. Table 4 summarizes those time measurements which show that one patient is scanned every 17 min on a SAFOV and 7–8 min on a LAFOV.

These results are extrapolated to the WT-TB-PET. Note that the design and system integration of a CT component for the WT-TB-PET is in the design phase and subject of future research. Hence, for now, the scout and CT scan time is assumed to be equal to that of the SAFOV/LAFOV. Patient positioning and transfer time for the WT-TB-PET were derived from the mock-up measurements. Finally, based on these numbers, we can derive an expected throughput of 89 patients per 8-h working day (one patient every 5 min) using the WT-TB-PET concept.

### Radiotracer cost comparisons

To estimate the cost of the radiotracer ([^18^F]FDG) with each scanner set-up, the required activity at the beginning of the day was determined (assuming an equal injected activity per patient of 185 MBq, 5 mCi). The requirement is that after injecting all patients (spread over the day), the remaining activity at the end of the day is still 1.85 GBq (50 mCi). Table 5 shows the efficiency improvements by injecting and scanning faster (more patients/hour) while the radiotracer decays. The WT-TB-PET requires 66% less ordered activity per patient (compared to a SAFOV PET scanner). The LAFOV (in full angle) requires 45% less ordered activity per patient.

**Table 5:** Estimates of radiotracer cost reduction. The activity is calculated to ensure all patients can be injected with 5 mCi and at end of day 1.85 GBq is left (to enable extra or delayed scans)

System concept	SAFOV	LAFOV (Limited angle)	LAFOV (Full angle)	WT-TB-PET BGO	WT-TB-PET LYSO
# scans on 8 h working day	28	53	60	73	86
Time in between scans (sec)	1020	537	475	394	334
Required activity at start of day in GBq	66,6	91.39	98.05	80.54	89,73
Reduction in radiotracer cost/patient (%)	0	37%	45%	60%	66%

Assuming that the cost of a commercial [^18^F]FDG radiotracer dose/patient is about 100 Euro/patient (this number is highly variable between locations and is based on the average in a region Belgium with a high density of cyclotrons), scanning 100 patients on SAFOV over multiple days would cost about 10,000 Euro (radiotracer only). In the new WT-TB-PET LYSO design, this radiotracer cost (for scanning 100 patients) would be reduced with 66% to 4400 Euro.

The number of 28 patients/day on a SAFOV leads to about 5600 patients on 200 working days. In this scenario, the total budget saved on [^18^F]FDG radiotracer per year is 56 × 6600 = 369.6 kEuro.

## Discussion

Current TB-PET scanners come at high acquisition and installation costs, which limits their implementation into more clinical use. However, with the ongoing and foreseen gradual changes in the patient population and clinical needs, PET imaging will play a prominent role, not only in detection but also increasingly in therapy prediction and follow-up. To accommodate the growing number of patients and demand for repeat scans, PET scanners should evolve towards achieving: (i) lower radiation dose, (ii) faster, and (iii) lower cost imaging (including system and procedure cost), in addition to (iv) less staff per scan. Moreover, for selected screening (now becoming more frequent, e.g., in lung cancer with CT), efficient patient throughput and low cost are important driving factors. In this aspect, the downside of the current TB-PET-CT systems is that the high acquisition and service costs are only partially compensated for by the limited improvement in the patient throughput owing to only the faster PET acquisition time; while the patient positioning, scout and CT time become the dominant and impeding factor in achieving faster imaging. Therefore, in this paper, we present a new design concept for TB-PET to go towards faster (and potentially also lower radiation dose) imaging with higher patient throughput while ensuring a lower component cost. As the limit in TB-PET-CT throughput becomes patient positioning on the bed, we, therefore, propose a flat panel TB-PET design with the patients standing upright: the walk-through TB-PET [23].

A PET design based on flat panels with the patient lying on the bed was already proposed in 1990 by Terry Jones at the first IEEE MIC Conference in Washington. Later in the 1990s, several groups worked on coincidence imaging with gamma cameras (based on suboptimal flat panel NaI detectors) rotating around the patient lying on a bed. Due to the low sensitivity, long acquisition times were required, and the image quality was suboptimal. For this reason, the reimbursement for these gamma camera PET systems was withdrawn [25–28]. Besides, several clinical imaging systems (like breast tomosynthesis, radiography and even CT) with patients in standing positions have been installed in hospitals [29–31]. In general, these systems lead to high throughput and improve patients’ experience by omitting the positioning on the bed. Similarly, upright MRI scanners [32] and cone beam CT systems have become commercially available.

As shown in several studies [33–37], the most recent PET detectors have become very fast and highly sensitive, which allows to have the patient in the (upright) standing position. High throughput can be achieved with a concept somewhat similar to that in security screening systems in airports (using mm-wave scanners and planar X-ray), which even claim a throughput of several people per minute. Such a walk-through TB-PET design allows to skip the time-consuming positioning of the patient as well as the acquisition time related to the scout view and CT scans. The proposed PET design aims to acquire sufficient PET data in a short time frame (motivated by the results of the Siemens Quadra and United Imaging Explorer) for a full torso scan (including the head of the patient). Very short acquisition time (< 10–20 s) can also enable breath-hold PET to minimize internal motion and ease registration to a previously acquired CT scan. This is still a subject of ongoing research. If required, deep learning (DL)-based noise reduction techniques can be applied to the reconstructed images to further optimize the acquisition time and dose [38, 39].

Another major benefit of this design is the use of monolithic detectors. These offer excellent intrinsic spatial resolution (range 1–1.5 mm), thereby outperforming classical pixelated detectors by a factor of 2–3, and provide a uniform spatial resolution over the FOV thanks to their DOI capabilities [18]. The TOF measurements on these detectors also indicate results quite close (327 ps) to existing pixelated L(Y)SO detectors (210–500 ps) already implemented on commercial clinical PET-CT systems. The low cost of the proposed WT-TB-PET scanner is particularly interesting for countries with limited access to PET imaging as these will often have a high patient load, limited radiotracer availability and limited installation space available. The two WT-TB-PET proposals with either BGO or L(Y)SO have each their own advantage: the lower cost system is the one with BGO and the higher throughput can be achieved with L(Y)SO (despite its lower sensitivity), both will have very comparable spatial resolution. Again, including both options illustrate the trade-offs with this design, both have benefits compared with current designs.

**Fig. 4:**
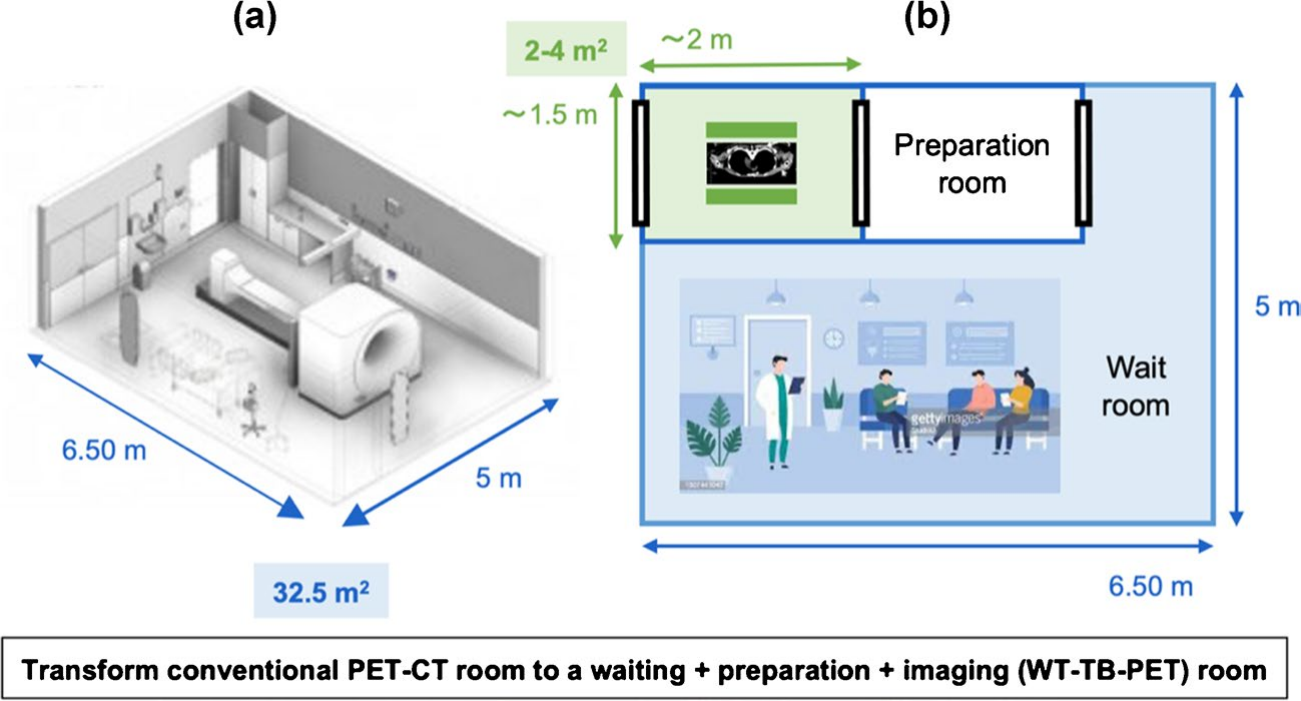
(**a**) Large installation space needed for conventional PET-CT scanner. (**b**) Compact footprint of the walk-through TB-PET enables to transform the PET-CT imaging room into a waiting room

The proposed WT-TB-PET design [23] is not the only possible candidate for reducing the cost of a TB-PET system to promote it towards more clinical use [40]. One alternative is a medium axial FOV scanner of about 60–70-cm axial length [41], for quickly scanning the body. For the same reasons, sparse long axial FOV PET designs [42–44] and systems based on lower cost components or plastic scintillators (e.g., J-PET [45, 46]) have also been proposed. However, the WT-TB-PET is the only design that maintains a high sensitivity with a smaller number of detectors. Owing to its upright design, it can effectively reduce the time required for patient positioning and ensure patient comfort. Shorter acquisitions are known to also reduce motion artifacts. Another major advantage of such a compact design is the small footprint required to install such a system. To illustrate this, we used the layout of a typical PET-CT room (with a conventional PET-CT scanner), removed the scanner, and rearranged the space (Fig. 4). The installation of the WT-TB-PET would only require about 2–6 m^2^ (compared to the 35–40 m^2^ installation spaces required for LAFOV) and can even enable the transformation of the scanner room into a patient waiting area. Please note that like in conventional PET-CT system also additional space for cooling will be required, but likely this can also be reduced compared to current LAFOV systems as the flat panel design has about a factor 2 less detector surface.

The disadvantage of not having a CT for attenuation or scatter correction seems nowadays relatively easily solved by using DL-based attenuation and scatter correction [47]. Alternatives include the use of transmission sources or MLAA reconstruction methods [48–51]. Such anatomical maps should enable easy registration with prior acquired (PET −)CT scans. This DL network incorporates the domain knowledge and is therefore robust in the application on new radiotracers and scanners, which has not been touched on in the training procedure. For proof-of-concept, this DL network was only trained on 470 subjects with [^18^F]FDG-PET scanned on Siemens Biograph Vision 450 but proved to be robust in the application of external scanners of United Imaging uMI 780, GE Discovery MI, and Siemens Biograph Vision 600 as well as of external radiotracers of [^68^ Ga]Ga-FAPI, [^68^ Ga]Ga-DOTA-TATE, [^68^ Ga]Ga-DOTA-TOC, and [^18^F]-PSMA[47]. The robustness of this DL network enhances the generality and flexibility of the proposed scanner.

CT is not only important for attenuation and scatter correction but plays also an important role in the reporting by physicians. This project first focusses first on the PET system but we have a similar relatively simple approach in development (still early stage) for a high-quality standing CT integrated in line with the WT-PET.

The novelty of the system approach presented here is the patient-centered design combined with two very sensitive flat panel detectors positioned close to the patient. If we consider PET imaging as a workflow process, it is quite clear that the PET acquisition time is only one of the factors determining the possible patient throughput. By switching from a patient positioning on a horizontal table to a patient standing in between two panels, it is evident that the throughput will increase: the patient does not need to be positioned on the table, nor transferred in and out of the scanner (before and after imaging). While this factor was not so important in the previous generation of PET-CT systems (long PET scan times of 15–20 min), it has become an important factor in the latest generation of PET scanners with high sensitivity. There has not been a proposed research or clinical system combining all these characteristics and addressing at the same time issues crucial to patients (comfort and fast scanning), hospitals (low cost and high throughput), and physicians (good-quality images).

Implementing such a system will evidently identify other bottlenecks in the workflow. The most evident one is the number of tracer uptake rooms. These will have to be modified to create more capacity, but as shown in the last figure extra space can be created in the imaging room. In general, the clinical space should be remodeled to ensure an optimal workflow and patient transfer. More scans also lead to more reporting time for physicians. It can be expected that in the future AI-aided reporting can increase the number of reported scans per physician. Although very short scan times become possible a standing position is not feasible for all patients. A certain fraction (estimated at < 10–20%, but dependant on local setting) of patients is bedridden. Therefore, an optimal combination would be a WT-PET with a SAFOV scanner.

The WT-PET does not exclude new applications, e.g., selected screening, brain–body interactions, low-dose scans which can also be done easier in a more patient-friendly way in this system. While in many cases a torso only scan is done, there is surely some value in including the brain (check for metastases) for some indications, but this may require more specific tracers. Here, it may also be interesting to further develop multi-tracer scans.

In the next step, we will first perform detailed system simulations and image reconstruction for this new scanner design for a range of patient sizes. There is evidently a challenge with the inevitable motion of the patient in a standing position. While our design aims at fast imaging (30 s to 1 min), motion estimation will be used to correct for the external patient motion. Tests will be performed in the mock-up to see whether breath-hold is feasible with 30-s acquisitions. The high sensitivity should enable the reconstruction of very short frames (1 s), which can be registered. A second mock-up version (with automatic laser-based height adjustment of both flat panels) will be constructed, and the detectors will be assembled into modules. Integration with a compact CT system is also a planned work in progress.

## Conclusions

TB-PET systems have the potential to scan much faster (and/or at lower radiation dose) but lack very high (efficient) patient throughput due to patient positioning and preparatory scans and have a high acquisition cost. The proposed walk-through TB-PET flat panel design (with 1.9 × less detector surface) based on patient sizes provides a solution to achieve much lower cost than current LAFOV scanners and close to SAFOV scanners. Three times higher patient throughput seems feasible with this design. This is technically achieved by combining a new geometry (based on two opposing flat panel detectors between which the patient stands in upright position) with the latest monolithic detector technology (to enable high resolution at close distance). For the proposed design, we consider the use of BGO to further reduce the cost of the most expensive component (= scintillator) in PET instrumentation or the use of LYSO to maximize performance (higher throughput thanks to better TOF). This finally results in a total budget for the scintillator and SiPM components of this new WT-TB-PET that is 1.2–2 × higher compared to a regular (SAFOV) and clearly lower than LAFOV PET-CT scanner, respectively. The WT design can provide > 3 × throughput at < 2 × cost (even with LYSO) compared to SAFOV systems. In contrast, the LAFOV systems with conventional geometry provide only 2 × throughout at 4 × cost compared to SAFOV system.
